# Effect of Atlas Vertebrae Realignment in Subjects with Migraine: An Observational Pilot Study

**DOI:** 10.1155/2015/630472

**Published:** 2015-12-10

**Authors:** H. Charles Woodfield, D. Gordon Hasick, Werner J. Becker, Marianne S. Rose, James N. Scott

**Affiliations:** ^1^Upper Cervical Research Foundation, 5353 Wayzata Boulevard, Suite 350, Minneapolis, MN 55416, USA; ^2^The Britannia Clinic, 5005 Elbow Drive SW No. 201, Calgary, AB, Canada T2S 2T6; ^3^University of Calgary and Alberta Health Services, Foothills Hospital, 1403 29 Street NW, Calgary, AB, Canada T2N 2T9; ^4^Rho Sigma Scientific Consultants, 119 Valencia Road NW, Calgary, AB, Canada T3A 2B7; ^5^Departments of Diagnostic Imaging and Clinical Neurosciences, University of Calgary, 2500 University Drive NW, Calgary, AB, Canada T2N 1N4

## Abstract

*Introduction*. In a migraine case study, headache symptoms significantly decreased with an accompanying increase in intracranial compliance index following atlas vertebrae realignment. This observational pilot study followed eleven neurologist diagnosed migraine subjects to determine if the case findings were repeatable at baseline, week four, and week eight, following a National Upper Cervical Chiropractic Association intervention. Secondary outcomes consisted of migraine-specific quality of life measures.* Methods*. After examination by a neurologist, volunteers signed consent forms and completed baseline migraine-specific outcomes. Presence of atlas misalignment allowed study inclusion, permitting baseline MRI data collection. Chiropractic care continued for eight weeks. Postintervention reimaging occurred at week four and week eight concomitant with migraine-specific outcomes measurement.* Results*. Five of eleven subjects exhibited an increase in the primary outcome, intracranial compliance; however, mean overall change showed no statistical significance. End of study mean changes in migraine-specific outcome assessments, the secondary outcome, revealed clinically significant improvement in symptoms with a decrease in headache days.* Discussion*. The lack of robust increase in compliance may be understood by the logarithmic and dynamic nature of intracranial hemodynamic and hydrodynamic flow, allowing individual components comprising compliance to change while overall it did not. Study results suggest that the atlas realignment intervention may be associated with a reduction in migraine frequency and marked improvement in quality of life yielding significant reduction in headache-related disability as observed in this cohort. Future study with controls is necessary, however, to confirm these findings. Clinicaltrials.gov registration number is NCT01980927.

## 1. Introduction

It has been proposed that a misaligned atlas vertebra creates spinal cord distortion disrupting neural traffic of brain stem nuclei in the medulla oblongata encumbering normal physiology [1–4].

The objective of the National Upper Cervical Chiropractic Association (NUCCA) developed atlas correction procedure is restoration of misaligned spinal structures to the vertical axis or gravity line. Described as the “restoration principle,” realignment aims to reestablish a patient's normal biomechanical relationship of the upper cervical spine to the vertical axis (gravity line). Restoration is characterized as being architecturally balanced, being capable of unrestricted range of motion, and allowing a significant decrease in gravitational stress [3]. The correction theoretically removes the cord distortion, created by an atlas misalignment or atlas subluxation complex (ASC), as specifically defined by NUCCA. Neurologic function is restored, specifically thought to be in the brain stem autonomic nuclei, which affect the cranial vascular system that includes Cerebrospinal Fluid (CSF) [3, 4].

The intracranial compliance index (ICCI) appears to be a more sensitive assessment of changes made in craniospinal biomechanical properties in symptomatic patients than the local hydrodynamic parameters of CSF flow velocities and cord displacement measurements [5]. Based on that information, previously observed relationships of increased intracranial compliance to marked reduction in migraine symptoms following atlas realignment provided incentive for using the ICCI as the study objective primary outcome.

ICCI affects the ability of the Central Nervous System (CNS) to accommodate physiologic volume fluctuations that occur, thereby avoiding ischemia of underlying neurologic structures [5, 6]. A state of high intracranial compliance enables any volume increase to occur in the intrathecal CNS space without causing an intracranial pressure increase that occurs primarily with arterial inflow during systole [5, 6]. Outflow occurs in the supine position via the internal jugular veins or when upright, via paraspinal or secondary venous drainage. This extensive venous plexus is valveless and anastomotic, allowing blood to flow in a retrograde direction, into the CNS through postural changes [7, 8]. Venous drainage plays an important role in regulating the intracranial fluid system [9]. Compliance appears to be functional and dependent on the free egress of blood via these extracranial venous drainage pathways [10].

Head and neck injury could create abnormal function of the spinal venous plexus that may impair spinal venous drainage, possibly because of autonomic dysfunction secondary to spinal cord ischemia [11]. This decreases accommodation of volume fluctuations within the cranium creating a state of decreased intracranial compliance.

Damadian and Chu describe return of a normal CSF outflow measured at mid-C-2, exhibiting a 28.6% reduction of the measured CSF pressure gradient in the patient where the atlas had been optimally realigned [12]. The patient reported freedom from symptoms (vertigo and vomiting when recumbent) consistent with the atlas remaining in alignment.

A hypertension study using the NUCCA intervention suggests a possible mechanism underlying the blood pressure decrease could be resultant from changes in cerebral circulation in relation to atlas vertebrae position [13]. Kumada et al. investigated a trigeminal-vascular mechanism in brain stem blood pressure control [14, 15]. Goadsby et al. have presented compelling evidence that migraine originates via a trigeminal-vascular system mediated through the brain stem and upper cervical spine [16–19]. Empirical observation reveals significant reduction of migraine patients' headache disability after application of the atlas correction. Using migraine-diagnosed subjects seemed ideal for investigating proposed cerebral circulation changes following atlas realignment as originally theorized in the hypertension study conclusions and seemingly supported by a possible brain stem trigeminal-vascular connection. This would further advance a developing working pathophysiologic hypothesis of atlas misalignment.

Results from an initial case study demonstrated substantial increase in ICCI with decrease in migraine headache symptoms following the NUCCA atlas correction. A 62-year-old male with neurologist diagnosed chronic migraine volunteered for a before-after intervention case study. Using Phase Contrast-MRI (PC-MRI), changes in cerebral hemodynamic and hydrodynamic flow parameters were measured at baseline, 72 hours, and then four weeks after the atlas intervention. The same atlas correction procedure used in the hypertension study was followed [13]. 72 hours after study revealed a noteworthy change in the intracranial compliance index (ICCI), from 9.4 to 11.5, to 17.5 by week four, after intervention. Observed changes in venous outflow pulsatility and predominant secondary venous drainage in the supine position warranted additional investigation further inspiring a study of migraine subjects in this case series.

The possible effects of the atlas misalignment or ASC on venous drainage are unknown. Careful examination of intracranial compliance in relation to effects of an atlas misalignment intervention may provide insight into how the correction might influence migraine headache.

Using PC-MRI, this current study's primary objective, and primary outcome, measured ICCI change from baseline to four and eight weeks following a NUCCA intervention in a cohort of neurologist selected migraine subjects. As observed in the case study, the hypothesis supposed that a subject's ICCI would increase following the NUCCA intervention with a corresponding decrease in migraine symptoms. If present, any observed changes in venous pulsatility and drainage route were to be documented for further comparison. To monitor migraine symptoms response, the secondary outcomes included patient reported outcomes to measure any related change in Health Related Quality of Life (HRQoL), similarly used in migraine research. Throughout the study, subjects maintained headache diaries documenting the decrease (or increase) in the number of headache days, intensity, and medication used.

Conducting this observational case series, pilot study, allowed for additional investigation into aforementioned physiologic effects in further development of a working hypothesis into the pathophysiology of an atlas misalignment. Data required for estimation of statistically significant subject sample sizes and resolving procedural challenges will provide needed information for developing a refined protocol to conduct a blinded, placebo controlled migraine trial using the NUCCA correction intervention.

## 2. Methods

This research maintained compliance with the Helsinki Declaration for research on human subjects. The University of Calgary and Alberta Health Services Conjoint Health Research Ethics Board approved the study protocol and subject informed consent form, Ethics ID: E-24116. ClinicalTrials.gov assigned the number NCT01980927 after registration of this study (https://clinicaltrials.gov/ct2/show/NCT01980927).

**Figure 1:**
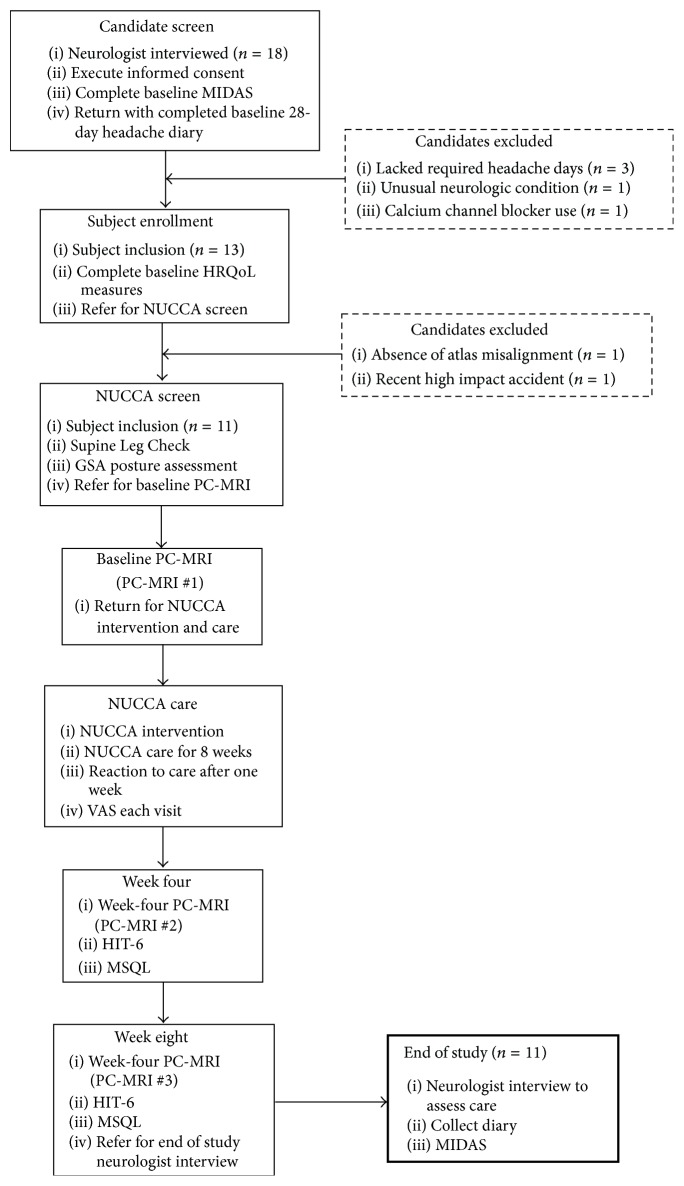
Subject disposition and study flow (*n* = 11). GSA: Gravity Stress Analyzer. HIT-6: Headache Impact Test-6. HRQoL: Health Related Quality of Life. MIDAS: Migraine Disability Assessment Scale. MSQL: Migraine-Specific Quality of Life Measure. NUCCA: National Upper Cervical Chiropractic Association. PC-MRI: Phase Contrast Magnetic Resonance Imaging. VAS: Visual Analog Scale.

Subject recruitment and screening occurred at the Calgary Headache Assessment and Management Program (CHAMP), a neurology-based specialist referral clinic (see Figure 1, Table 1). CHAMP evaluates patients resistant to standard pharmacotherapy and medical treatment for migraine headache that no longer provides migraine symptom relief. Family and primary care physicians referred potential study subjects to CHAMP making advertising unnecessary.

Study inclusion required volunteers, between the ages of 21 and 65 years, that satisfy specific diagnostic criteria for migraine headache. A neurologist with several decades of migraine experience screened applicants utilizing the International Classification of Headache Disorders (ICHD-2) for study inclusion [20]. Potential subjects, naïve to upper cervical chiropractic care, must have demonstrated through self-report between ten and twenty-six headache days per month over the previous four months. At least eight headache days per month had to reach an intensity of at least four on a zero to ten VAS pain scale, unless treated successfully with a migraine-specific medication. At least four separate headache episodes per month separated by at least a 24-hour pain-free interval were required.

Significant head or neck trauma occurring within one year prior to study entry excluded candidates. Further exclusion criteria included acute medication overuse, a history of claustrophobia, cardiovascular or cerebrovascular disease, or any CNS disorder other than migraine. Table 1 describes the complete inclusion and exclusion criteria considered. Using an experienced board certified neurologist to screen potential subjects while adhering to the ICHD-2 and guided by the inclusion/exclusion criteria, the exclusion of subjects with other sources of headache such as muscular tension and medication overuse rebound headache would increase the likelihood of successful subject recruitment.

Those meeting initial criteria signed informed consent and then completed a baseline Migraine Disability Assessment Scale (MIDAS). The MIDAS requires twelve weeks to demonstrate clinically significant change [21]. This allowed adequate time to pass to discern any possible changes. Over the next 28 days, candidates recorded a headache diary providing baseline data while confirming the number of headache days and intensity required for inclusion. After the four weeks, the diary check diagnostic substantiation permitted administration of remaining baseline HRQoL measures:

1. Migraine-Specific Quality of Life Measure (MSQL) [22],
2. Headache Impact Test-6 (HIT-6) [23],
3. subject current global assessment of headache pain (VAS).

Referral to the NUCCA practitioner, to determine presence of atlas misalignment, confirmed need for intervention finalizing a subject's study inclusion∖exclusion. Absence of atlas misalignment indicators excluded candidates. After scheduling appointments for NUCCA intervention and care, qualified subjects obtained baseline PC-MRI measures. Figure 1 summarizes subject disposition throughout the study.

The initial NUCCA intervention required three consecutive visits: (1) Day One, atlas misalignment assessment, before-correction radiographs; (2) Day Two, NUCCA correction with after-correction assessment with radiographs; and (3) Day Three, after-correction reassessment. Follow-up care occurred weekly for four weeks, then every two weeks for the remainder of the study period. At each NUCCA visit, subjects completed a current assessment of headache pain (please rate your headache pain on average over the past week) using a straight edge and pencil in marking a 100 mm line (VAS). One week after the initial intervention, subjects completed a “Possible Reaction to Care” questionnaire. This assessment has past been used for successfully monitoring adverse events related to various upper cervical correction procedures [24].

At week four, PC-MRI data were obtained and subjects completed an MSQL and HIT-6. End of study PC-MRI data were collected at week eight followed by a neurologist exit interview. Here, subjects completed final MSQOL, HIT-6, MIDAS, and VAS outcomes and headache diaries were collected.

At the week-8 neurologist visit, two willing subjects were offered a long-term follow-up opportunity for a total study period of 24 weeks. This involved further NUCCA reassessment monthly for 16 weeks after completion of the initial 8-week study. The purpose of this follow-up was to help determine if headache improvement continued contingent upon maintenance of atlas alignment while observing for any long-term effect of NUCCA care on ICCI. Subjects desiring to participate signed a second informed consent for this phase of study and continued monthly NUCCA care. At the end of 24 weeks from the original atlas intervention, the fourth PC-MRI imaging study occurred. At the neurologist exit interview, final MSQOL, HIT-6, MIDAS, and VAS outcomes and headache diaries were collected.

**Figure 2:**
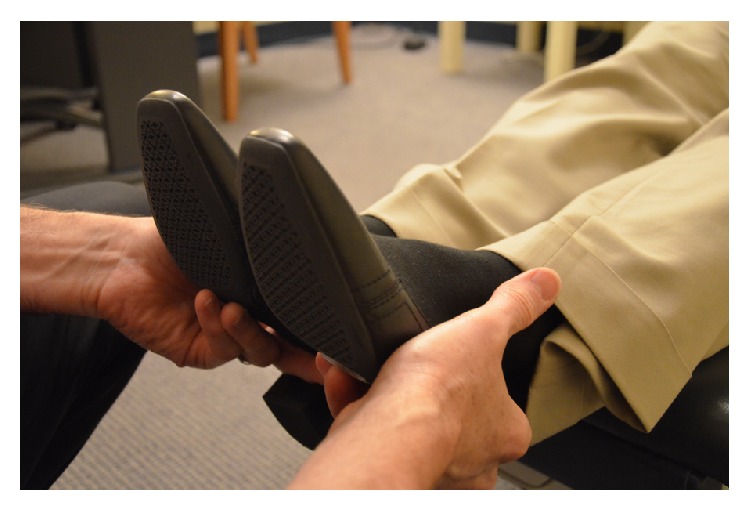
Supine Leg Check Screening Test (SLC). Observation of an apparent “short leg” indicates possible atlas misalignment. These appear even.

**Figure 3:**
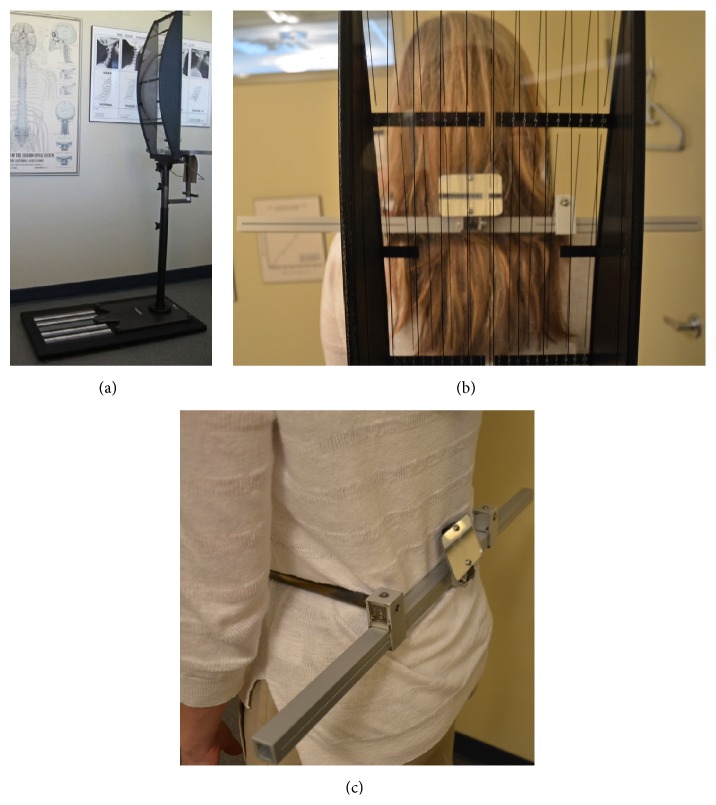
Gravity Stress Analyzer (GSA). (a) Device determines postural asymmetry as a further indicator of atlas misalignment. Positive findings in the SLC and GSA indicate need for NUCCA radiographic series. (b) Balanced patient with no postural asymmetry. (c) Hip calipers used to measure pelvis asymmetry.

**Figure 4:**
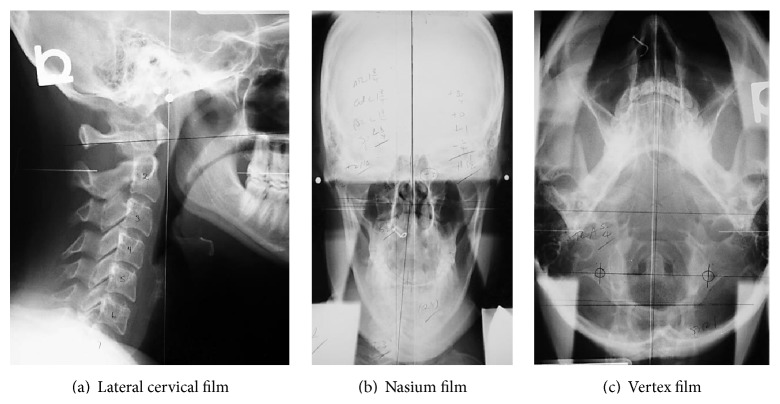
NUCCA radiograph series. These films are used to determine atlas misalignment and developing a correction strategy. After-correction radiographs or postfilms ensure the best correction has been made for that subject.

**Figure 5:**
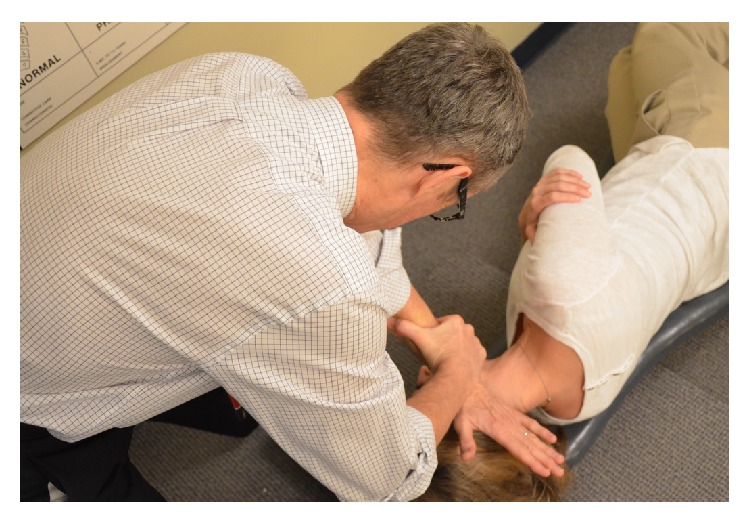
Making a NUCCA correction. The NUCCA practitioner delivers a triceps pull adjustment. The practitioner's body and hands are aligned to deliver an atlas correction along an optimal force vector using information obtained from radiographs.

The same NUCCA procedure as previously reported was followed using the established protocol and standards of care developed through NUCCA Certification for assessment and atlas realignment or correction of the ASC (see Figures 2–5) [2, 13, 25]. Assessment for the ASC includes screening for functional leg-length inequality with the Supine Leg Check (SLC) and examination of postural symmetry using the Gravity Stress Analyzer (Upper Cervical Store, Inc., 1641 17 Avenue, Campbell River, BC, Canada V9W 4L5) (see Figures 2 and 3(a)–3(c)) [26–28]. If SLC and postural imbalances are detected, a three-view radiographic exam is indicated to determine the multidimensional orientation and degree of craniocervical misalignment [29, 30]. A thorough radiographic analysis provides information to determine a subject specific, optimal atlas correction strategy. The clinician locates anatomic landmarks from the three-view series, measuring structural and functional angles that have deviated from established orthogonal standards. The degree of misalignment and atlas orientation are then revealed in three dimensions (see Figures 4(a)–4(c)) [2, 29, 30]. Radiographic equipment alignment, reduction of collimator port size, high-speed film-screen combinations, special filters, specialized grids, and lead shielding minimize subject radiation exposure. For this study, average total measured Entrance Skin Exposure to subjects from the before-after-correction radiographic series was 352 millirads (3.52 millisieverts).

The NUCCA intervention involves a manual correction of the radiographically measured misalignment in the anatomical structure between the skull, atlas vertebra, and cervical spine. Utilizing biomechanical principles based on a lever system, the doctor develops a strategy for proper

1. subject positioning,
2. practitioner stance,
3. force vector to correct the atlas misalignment.

Subjects are placed on a side-posture table with the head specifically braced using a mastoid support system. Application of the predetermined controlled force vector for the correction realigns the skull to the atlas and neck to the vertical axis or center of gravity of the spine. These corrective forces are controlled in depth, direction, velocity, and amplitude, producing an accurate and precise reduction of the ASC.

Using the pisiform bone of the contact hand, the NUCCA practitioner contacts the atlas transverse process. The other hand encircles the wrist of the contact hand, to control the vector while maintaining the depth of force generated in application of the “triceps pull” procedure (see Figure 5) [3]. By understanding spinal biomechanics, the practitioner's body and hands are aligned to produce an atlas correction along the optimal force vector. The controlled, nonthrusting force is applied along the predetermined reduction pathway. It is specific in its direction and depth to optimize the ASC reduction assuring no activation in the reactive forces of the neck muscles in response to the biomechanical change. It is understood that an optimal reduction of the misalignment promotes long-term maintenance and stability of spinal alignment.

Following a short rest period, an after-assessment procedure, identical to the initial evaluation, is performed. A postcorrection radiograph examination uses two views to verify return of the head and cervical spine into optimum orthogonal balance. Subjects are educated in ways to preserve their correction, thus preventing another misalignment.

Subsequent NUCCA visits were comprised of headache diary checks and a current assessment of headache pain (VAS). Leg length inequality and excessive postural asymmetry were used in determining the need for another atlas intervention. The objective for optimal improvement is for the subject to maintain the realignment for as long as possible, with the fewest number of atlas interventions.

In a PC-MRI sequence, contrast media are not used. PC-MRI methods collected two data sets with different amounts of flow sensitivity acquired by relating gradient pairs, which sequentially dephase and rephase spins during the sequence. The raw data from the two sets are subtracted to calculate a flow rate.

An on-site visit by the MRI Physicist provided training for the MRI Technologist and a data transfer procedure was established. Several practice scans and data transfers were performed to ensure data collection succeeded without challenges. A 1.5-tesla GE 360 Optima MR scanner (Milwaukee, WI) at the study imaging center (EFW Radiology, Calgary, Alberta, Canada) was used in imaging and data collection. A 12-element phased array head coil, 3D magnetization-prepared rapid-acquisition gradient echo (MP-RAGE) sequence was used in anatomy scans. Flow sensitive data were acquired using a parallel acquisition technique (iPAT), acceleration factor 2.

To measure blood flow to and from the skull base, two retrospectively gated, velocity-encoded cine-phase-contrast scans were performed as determined by individual heart rate, collecting thirty-two images over a cardiac cycle. A high-velocity encoding (70 cm/s) quantified high-velocity blood flow perpendicular to the vessels at the C-2 vertebra level includes the internal carotid arteries (ICA), vertebral arteries (VA), and internal jugular veins (IJV). Secondary venous flow data of vertebral veins (VV), epidural veins (EV), and deep cervical veins (DCV) were acquired at the same height using a low-velocity encoding (7–9 cm/s) sequence.

Subject data were identified by Subject Study ID and imaging study date. The study neuroradiologist reviewed MR-RAGE sequences to rule out exclusionary pathologic conditions. Subject identifiers were then removed and assigned a coded ID permitting transfer via a secured tunnel IP protocol to the physicist for analysis. Using proprietary software volumetric blood, Cerebrospinal Fluid (CSF) flow rate waveforms and derived parameters were determined (MRICP version 1.4.35 Alperin Noninvasive Diagnostics, Miami, FL).

Using the pulsatility-based segmentation of lumens, time-dependent volumetric flow rates were calculated by integrating the flow velocities inside the luminal cross-sectional areas over all thirty-two images. Mean flow rates were obtained for the cervical arteries, primary venous drainage, and secondary venous drainage pathways. Total cerebral blood flow was obtained by summation of these mean flow rates.

A simple definition of compliance is a ratio of volume and pressure changes. Intracranial compliance is calculated from the ratio of the maximal (systolic) intracranial volume change (ICVC) and pressure fluctuations during the cardiac cycle (PTP-PG). Change in ICVC is obtained from momentary differences between volumes of blood and CSF entering and exiting the cranium [5, 31]. Pressure change during the cardiac cycle is derived from the change in the CSF pressure gradient, which is calculated from the velocity-encoded MR images of the CSF flow, using the Navier-Stokes relationship between derivatives of velocities and the pressure gradient [5, 32]. An intracranial compliance index (ICCI) is calculated from the ratio of ICVC and pressure changes [5, 31–33].

Statistical analysis considered several elements. ICCI data analysis involved a one-sample Kolmogorov-Smirnov test revealing a lack of normal distribution in the ICCI data, which were therefore described using the median and interquartile range (IQR). Differences between baseline and follow-up were to be examined using a paired* t*-test.

NUCCA assessments data were described using mean, median, and interquartile range (IQR). Differences between baseline and follow-up were examined using a paired* t*-test.

Depending on the outcome measure, baseline, week four, week eight, and week twelve (MIDAS only) follow-up values were described using the mean and standard deviation. MIDAS data collected at initial neurologist screening had one follow-up score at the end of twelve weeks.

Differences from baseline to each follow-up visit were tested using a paired* t*-test. This resulted in numerous *p* values from two follow-up visits for each outcome except the MIDAS. Since one purpose of this pilot is to provide estimates for future research, it was important to describe where differences occurred, rather than to use a one-way ANOVA to arrive at a single *p* value for each measure. The concern with such multiple comparisons is the increase in Type I error rate.

To analyze the VAS data, each subject scores were examined individually and then with a linear regression line that adequately fits the data. Use of a multilevel regression model with both random intercepts and random slope provided an individual regression line fitted for each patient. This was tested against a random intercept-only model, which fits a linear regression line with a common slope for all subjects, while intercept terms are allowed to vary. The random coefficient model was adopted, as there was no evidence that random slopes significantly improved the fit to the data (using a likelihood ratio statistic). To illustrate the variation in the intercepts but not in the slope, the individual regression lines were graphed for each patient with an imposed average regression line on top.

## 3. Results

From initial neurologist screening, eighteen volunteers were eligible for inclusion. After completion of baseline headache diaries, five candidates did not meet inclusion criteria. Three lacked the required headache days on baseline diaries to be included, one had unusual neurological symptoms with persistent unilateral numbness, and another was taking a calcium channel blocker. The NUCCA practitioner found two candidates ineligible: one lacking an atlas misalignment and the second with a Wolff-Parkinson-White condition and severe postural distortion (39°) with recent involvement in a severe high impact motor vehicle accident with whiplash (see Figure 1).

Eleven subjects, eight females and three males, average age forty-one years (range 21–61 years), qualified for inclusion. Six subjects presented chronic migraine, reporting fifteen or more headache days a month, with a total eleven-subject mean of 14.5 headache days a month. Migraine symptom duration ranged from two to thirty-five years (mean twenty-three years). All medications were maintained unchanged for the study duration to include their migraine prophylaxis regimens as prescribed.

Per exclusion criteria, no subjects included received a diagnosis of headache attributed to traumatic injury to the head and neck, concussion, or persistent headache attributed to whiplash. Nine subjects reported a very remote past history, greater than five years or more (average of nine years) prior to neurologist screen. This included sports-related head injuries, concussion, and/or whiplash. Two subjects indicated no prior head or neck injury (see Table 2).

Individually, five subjects demonstrated an increase in ICCI, three subject's values remained essentially the same, and three showed a decrease from baseline to end of study measurements. Overall changes in intracranial compliance are seen in Table 2 and Figure 8. The median (IQR) values of ICCI were 5.6 (4.8, 5.9) at baseline, 5.6 (4.9, 8.2) at week four, and 5.6 (4.6, 10.0) at week eight. Differences were not statistically different. The mean difference between baseline and week four was −0.14 (95% CI −1.56, 1.28), *p* = 0.834, and between baseline and week eight was 0.93 (95% CI −0.99, 2.84), *p* = 0.307. These two subject's 24-week ICCI study results are seen in Table 6. Subject 01 displayed an increasing trend in ICCI from 5.02 at baseline to 6.69 at week 24, whereas at week 8, results were interpreted as consistent or remaining the same. Subject 02 demonstrated a decreasing trend in ICCI from baseline of 15.17 to 9.47 at week 24.

Table 3 reports changes in NUCCA assessments. The mean difference from before to after the intervention is as follows: (1) SLC: 0.73 inches, 95% CI (0.61, 0.84) (*p* < 0.001); (2) GSA: 28.36 scale points, 95% CI (26.01, 30.72) (*p* < 0.001); (3) Atlas Laterality: 2.36 degrees, 95% CI (1.68, 3.05) (*p* < 0.001); and (4) Atlas Rotation: 2.00 degrees, 95% CI (1.12, 2.88) (*p* < 0.001). This would indicate that a probable change occurred following the atlas intervention as based on subject assessment.

**Figure 6:**
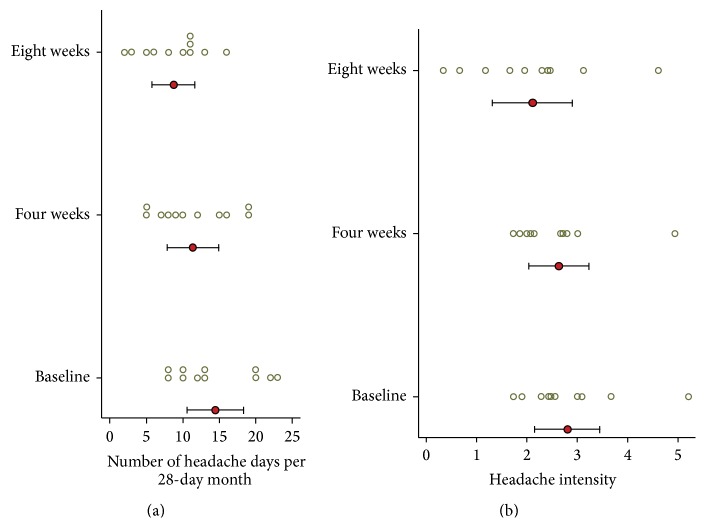
Headache days and headache pain intensity from diary (*n* = 11). (a) Number of headache days per month. (b) Average headache intensity (on headache days). Circle indicates the mean and the bar indicates the 95% CI. Circles are individual subject scores. A significant decrease in headache days per month was noticed at four weeks, almost doubling at eight weeks. Four subjects (#4, 5, 7, and 8) exhibited a greater than 20% decrease in headache intensity. Concurrent medication use may explain the small decrease in headache intensity.

Headache diary results are reported in Table 4 and Figure 6. At baseline subjects had mean 14.5 (SD = 5.7) headache days per 28-day month. During the first month following NUCCA correction, mean headache days per month decreased by 3.1 days from baseline, 95% CI (0.19, 6.0), *p* = 0.039, to 11.4. During the second month headache days decreased by 5.7 days from baseline, 95% CI (2.0, 9.4), *p* = 0.006, to 8.7 days. At week eight, six of the eleven subjects had a reduction of >30% in headache days per month. Over 24 weeks, subject 01 reported essentially no change in headache days while subject 02 had a reduction of one headache day a month from study baseline of seven to end of study reports of six days.

At baseline, mean headache intensity on days with headache, on a scale of zero to ten, was 2.8 (SD = 0.96). Mean headache intensity showed no statistically significant change at four (*p* = 0.604) and eight (*p* = 0.158) weeks. Four subjects (#4, 5, 7, and 8) exhibited a greater than 20% decrease in headache intensity.

Quality of life and headache disability measures are seen in Table 4. The mean HIT-6 score at baseline was 64.2 (SD = 3.8). At week four after NUCCA correction, mean decrease in scores was 8.9, 95% CI (4.7, 13.1), *p* = 0.001. Week-eight scores, compared to baseline, revealed mean decrease by 10.4, 95% CI (6.8, 13.9), *p* = 0.001. In the 24-week group, subject 01 showed a decrease of 10 points from 58 at week 8 to 48 at week 24 while subject 02 decreased 7 points from 55 at week 8 to 48 at week 24 (see Figure 9).

MSQL mean baseline score was 38.4 (SD = 17.4). At week four after correction, mean scores for all eleven subjects increased (improved) by 30.7, 95% CI (22.1, 39.2), *p* < 0.001. By week eight, end of study, mean MSQL scores had increased from baseline by 35.1, 95% CI (23.1, 50.0), *p* < 0.001, to 73.5. The follow-up subjects continued to show some improvement with increasing scores; however, many scores plateaued remaining the same since week 8 (see Figures 10(a)–10(c)).

Mean MIDAS score at baseline was 46.7 (SD = 27.7). At two months after NUCCA correction (three months following baseline), the mean decrease in subject's MIDAS scores was 32.1, 95% CI (13.2, 51.0), *p* = 0.004. The follow-up subjects continued to show improvement with decreasing scores with intensity showing minimal improvement (see Figures 11(a)–11(c)).

**Figure 7:**
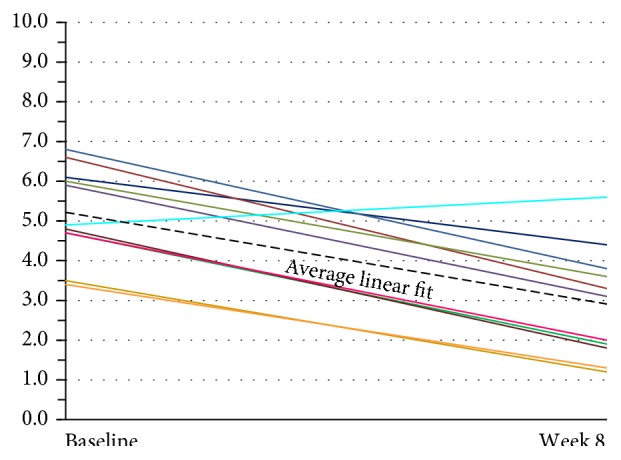
Subject global assessment of headache (VAS) (*n* = 11). There was substantial variation in baseline scores across these patients. The lines show individual linear fit for each of eleven patients. The thick dotted black line represents the average linear fit across all eleven patients. VAS: Visual Analog Scale.

Assessment of current headache pain from VAS scale data is seen in Figure 7. The multilevel linear regression model showed evidence of a random effect for the intercept (*p* < 0.001) but not for the slope (*p* = 0.916). Thus, the adopted random intercept model estimated a different intercept for each patient but a common slope. The estimated slope of this line was −0.044, 95% CI (−0.055, −0.0326), *p* < 0.001, indicating that there was a significant decrease in the VAS score of 0.44 per 10 days after baseline (*p* < 0.001). The mean baseline score was 5.34, 95% CI (4.47, 6.22). The random effects analysis showed substantial variation in the baseline score (SD = 1.09). As the random intercepts are normally distributed, this indicates that 95% of such intercepts lie between 3.16 and 7.52 providing evidence of substantial variation in the baseline values across patients. VAS scores continued showing improvement in the 24-week two-subject follow-up group (see Figure 12).

The most obvious reaction to the NUCCA intervention and care reported by ten subjects was mild neck discomfort, rated an average of three out of ten on pain assessment. In six subjects, pain began more than twenty-four hours after the atlas correction, lasting more than twenty-four hours. No subject reported any significant effect on their daily activities. All subjects reported satisfaction with NUCCA care after one week, median score, ten, on a zero to ten rating scale.

## 4. Discussion 

In this limited cohort of eleven migraine subjects, there was no statistically significant change in ICCI (primary outcome) after the NUCCA intervention. However, a significant change in HRQoL secondary outcomes did occur as summarized in Table 5. The consistency in the magnitude and direction of improvement across these HRQoL measures indicates confidence in enhancement of headache health over the two-month study following the 28-day baseline period.

Based on the case study results, this investigation hypothesized a significant increase in ICCI after the atlas intervention which was not observed. Use of PC-MRI allows quantification of the dynamic relationship between arterial inflow, venous outflow, and CSF flow between the cranium and the spinal canal [33]. Intracranial compliance index (ICCI) measures the brain's ability to respond to incoming arterial blood during systole. Interpretation of this dynamic flow is represented by a monoexponential relationship existing between CSF volume and CSF pressure. With increased or higher intracranial compliance, also defined as good compensatory reserve, the incoming arterial blood can be accommodated by the intracranial contents with a smaller change in intracranial pressure. While a change in intracranial volume or pressure could occur, based on the exponential nature of the volume-pressure relationship, a change in after-intervention ICCI may not be realized. An advanced analysis of the MRI data and further study are required for pinpointing practical quantifiable parameters to use as an objective outcome sensitive for documenting a physiologic change following atlas correction.

Koerte et al. reports of chronic migraine patients demonstrate a significantly higher relative secondary venous drainage (paraspinal plexus) in the supine position when compared to age- and gender-matched controls [34]. Four study subjects exhibited a secondary venous drainage with three of those subjects demonstrating notable increase in compliance after intervention. The significance is unknown without further study. Similarly, Pomschar et al. reported that subjects with mild traumatic brain injury (mTBI) demonstrate an increased drainage through the secondary venous paraspinal route [35]. The mean intracranial compliance index appears significantly lower in the mTBI cohort when compared to controls.

**Figure 8:**
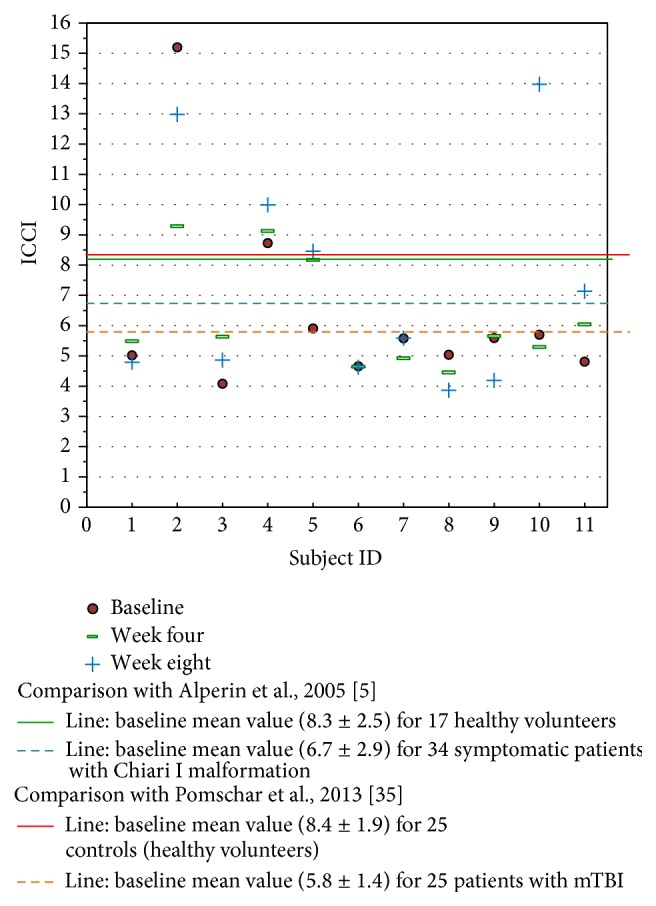
Study ICCI data compared to previously reported data in the literature. The MRI time values are fixed at baseline, week 4, and week 8 after intervention. This study's baseline values fall similar to the data reported by Pomschar on subjects presenting only with mTBI.

**Figure 9:**
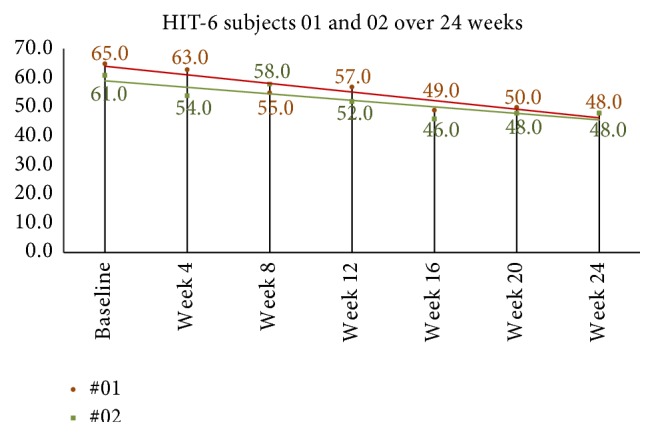
24-week HIT-6 scores in long-term follow-up subjects. Monthly scores continued to decrease after week 8, end of first study. Based on Smelt et al. criteria, it can be interpreted that a within-person minimally important change occurred between week 8 and week 24. HIT-6: Headache Impact Test-6.

Some perspective may be gained in comparison of this study's ICCI data to previously reported normal subjects and those with mTBI seen in Figure 8 [5, 35]. Limited by the small number of subjects studied, the significance these study's findings may have in relation to Pomschar et al. remains unknown, offering only speculation of possibilities for future exploration. This is further complicated by the inconsistent ICCI change observed in the two subjects followed for 24 weeks. Subject two with a secondary drainage pattern exhibited a decrease in ICCI following intervention. A larger placebo controlled trial with a statistically significant subject sample size could possibly demonstrate a definitive objectively measured physiologic change after application of the NUCCA correction procedure.

HRQoL measures are used clinically to assess the effectiveness of a treatment strategy to decrease pain and disability related to migraine headache. It is expected that an effective treatment improves patient perceived pain and disability measured by these instruments. All HRQoL measures in this study demonstrated significant and substantial improvement by week four following the NUCCA intervention. From week four to week eight only small improvements were noted. Again, only small improvements were noted in the two subjects followed for 24 weeks. While this study was not intended to demonstrate causation from the NUCCA intervention, the HRQoL results create compelling interest for further study.

From the headache diary, a significant decrease in headache days per month was noticed at four weeks, almost doubling at eight weeks. However, significant differences in headache intensity over time were not discernable from this diary data (see Figure 5). While the number of headaches decreased, subjects still used medication to maintain headache intensity at tolerable levels; hence, it is supposed that a statistically significant difference in headache intensity could not be determined. Consistency in the headache day numbers occurring in week 8 in the follow-up subjects could guide future study focus in determining when maximum improvement occurs to help in establishing a NUCCA standard of migraine care.

Clinically relevant change in the HIT-6 is important for completely understanding observed outcomes. A clinically meaningful change for an individual patient has been defined by the HIT-6 user guide as ≥5 [36]. Coeytaux et al., using four different analysis methods, suggest that a between-group difference in HIT-6 scores of 2.3 units over time may be considered clinically significant [37]. Smelt et al. studied primary care migraine patient populations in developing suggested recommendations using HIT-6 score changes for clinical care and research [38]. Dependent on consequences resulting from false positives or negatives, within-person minimally important change (MIC) using a “mean change approach” was estimated to be 2.5 points. When using the “receiver operating characteristic (ROC) curve analysis” a 6-point change is needed. Recommended between-group minimally important difference (MID) is 1.5 [38].

**Figure 10:**
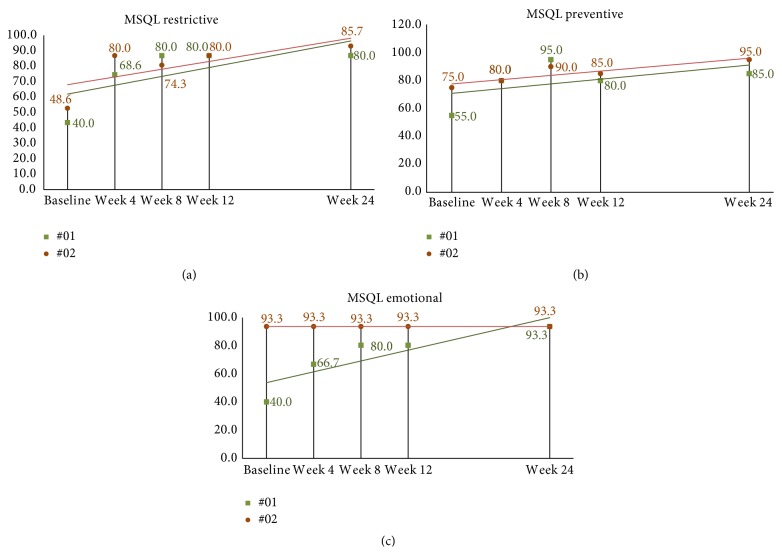
((a)–(c)) 24-week MSQL scores in long-term follow-up subjects. (a) Subject 01 has essentially plateaued after week 8 throughout to end of the second study. Subject 02 shows scores increasing over time demonstrating minimally important differences based on Cole et al. criteria by week 24. (b) Subject scores seem to peak by week 8 with both subjects showing similar scores reported at week 24. (c) Subject 2 scores remain consistent throughout the study while subject 01 shows steady improvement from baseline to the end of week 24. MSQL: Migraine-Specific Quality of Life Measure.

Using the “mean change approach,” all subjects but one reported a change (decrease) greater than −2.5. The “ROC analyses” also demonstrated improvement by all subjects but one. This “one subject” was a different person in each comparison analysis. Based on Smelt et al. criteria, the follow-up subjects continued to demonstrate within-person minimally important improvement as seen in Figure 10.

**Figure 11:**
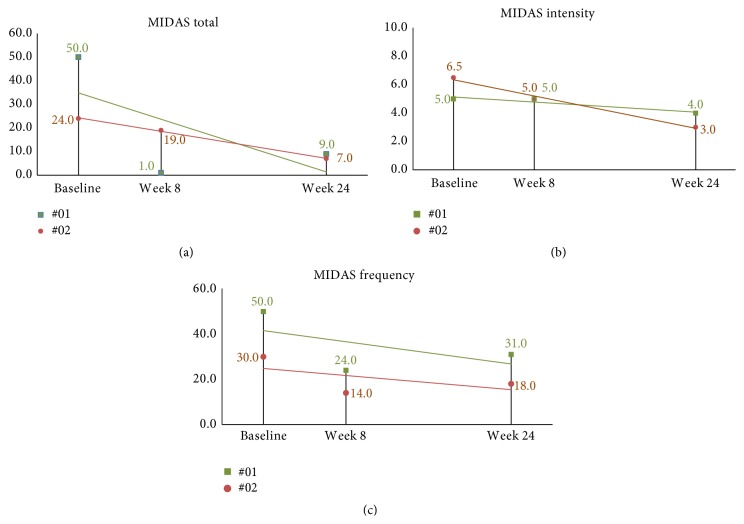
24-week MIDAS scores in long-term follow-up subjects. (a) Total MIDAS scores continued a decreasing trend over the 24-week study period. (b) Intensity scores continued improvement. (c) While 24-week frequency was higher than at week 8, improvement is observed when compared to baseline. MIDAS: Migraine Disability Assessment Scale.

All subjects but two showed improvement on the MIDAS score between baseline and three-month results. The magnitude of the change was proportional to the baseline MIDAS score, with all subjects but three reporting an overall fifty percent or greater change. The follow-up subjects continued to show improvement as seen in continued decrease in scores by week 24; see Figures 11(a)–11(c).

Use of the HIT-6 and MIDAS together as a clinical outcome may provide a more complete assessment of headache-related disability factors [39]. The differences between the two scales can predict disability from headache pain intensity and headache frequency, by providing more information on factors related to the reported changes than either outcome used alone. While the MIDAS appears to change more by headache frequency, headache intensity seems to affect HIT-6 score more than the MIDAS [39].

How migraine headache affects and limits patient perceived daily functioning is reported by the MSQL v. 2.1, across three 3 domains: role restrictive (MSQL-R), role preventive (MSQL-P), and emotional functioning (MSQL-E). An increase in scores indicates improvement in these areas with values ranging from 0 (poor) to 100 (best).

MSQL scales reliability evaluation by Bagley et al. report results to be moderately to highly correlated with HIT-6 (*r* = −0.60 to −0.71) [40]. Study by Cole et al. reports minimally important differences (MID) clinical change for each domain: MSQL-R = 3.2, MSQL-P = 4.6, and MSQL-E = 7.5 [41]. Results from the topiramate study report individual minimally important clinical (MIC) change: MSQL-R = 10.9, MSQL-P = 8.3, and MSQL-E = 12.2 [42].

All subjects except one experienced an individual minimally important clinical change for MSQL-R of greater than 10.9 by the week-eight follow-up in MSQL-R. All but two subjects reported changes of more than 12.2 points in MSQL-E. Improvement in MSQL-P scores increased by ten points or more in all subjects.

**Figure 12:**
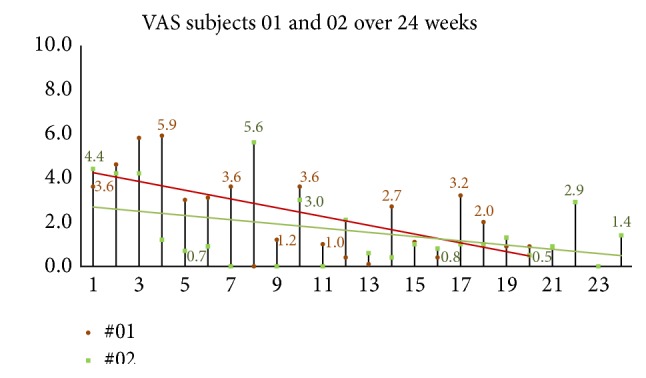
24-week follow-up group global assessment of headache (VAS). When subjects were queried, “*please rate your headache pain on average over the past week*” VAS scores continued showing improvement in the 24-week two-subject follow-up group.

Regression analysis of VAS ratings over time showed a significant linear improvement over the 3-month period. There was substantial variation in baseline scores across these patients. Little to no variation was observed in the rate of improvement. This trend appears to be the same in the subjects studied for 24 weeks as seen in Figure 12.

Many studies using pharmaceutical intervention have shown a substantial placebo effect in patients from migrainous populations [43]. Determining possible migraine improvement over six months, using another intervention as well as no intervention, is important for any comparison of results. The investigation into placebo effects generally accepts that placebo interventions do provide symptomatic relief but do not modify pathophysiologic processes underlying the condition [44]. Objective MRI measures may help in revealing such a placebo effect by demonstrating a change in physiologic measurements of flow parameters occurring after a placebo intervention.

Use of a three-tesla magnet for MRI data collection would increase the reliability of the measurements by increasing the amount of data used to make the flow and ICCI calculations. This is one of the first investigations using change in ICCI as an outcome in evaluating an intervention. This creates challenges in interpretation of MRI acquired data to base conclusions or further hypothesis development. Variability in relationships between blood flow to and from the brain, CSF flow, and heart rate of these subject-specific parameters has been reported [45]. Variations observed in a small three-subject repeated measures study have led to conclusions that information gathered from individual cases be interpreted with caution [46].

The literature further reports in larger studies significant reliability in collecting these MRI acquired volumetric flow data. Wentland et al. reported that measurements of CSF velocities in human volunteers and of sinusoidally fluctuating phantom velocities did not differ significantly between two MRI techniques used [47]. Koerte et al. studied two cohorts of subjects imaged in two separate facilities with different equipment. They reported that intraclass correlation coefficients (ICC) demonstrated a high intra- and interrater reliability of PC-MRI volumetric flow rate measurements remaining independent of equipment used and skill-level of the operator [48]. While anatomic variation exists between subjects, it has not prevented studies of larger patient populations in describing possible “normal” outflow parameters [49, 50].

Being based solely on patient subjective perceptions, there are limitations in using patient reported outcomes [51]. Any aspect affecting a subject's perception in their quality of life is likely to influence the outcome of any assessment used. Lack of outcome specificity in reporting symptoms, emotions, and disability also limits interpretation of results [51].

Imaging and MRI data analysis costs precluded use of a control group, limiting any generalizability of these results. A larger sample size would allow for conclusions based on statistical power and reduced Type I error. Interpretation of any significance in these results, while revealing possible trends, remains speculation at best. The big unknown persists in the likelihood that these changes are related to the intervention or to some other effect unknown to the investigators. These results do add to the body of knowledge of previously unreported possible hemodynamic and hydrodynamic changes after a NUCCA intervention, as well as changes in migraine HRQoL patient reported outcomes as observed in this cohort.

The values of collected data and analyses are providing information required for estimation of statistically significant subject sample sizes in further study. Resolved procedural challenges from conducting the pilot allow for a highly refined protocol to successfully accomplish this task.

In this study, the lack of robust increase in compliance may be understood by the logarithmic and dynamic nature of intracranial hemodynamic and hydrodynamic flow, allowing individual components comprising compliance to change while overall it did not. An effective intervention should improve subject perceived pain and disability related to migraine headache as measured by these HRQoL instruments used. These study results suggest that the atlas realignment intervention may be associated with reduction in migraine frequency, marked improvement in quality of life yielding significant reduction in headache-related disability as observed in this cohort. The improvement in HRQoL outcomes creates compelling interest for further study, to confirm these findings, especially with a larger subject pool and a placebo group.

## Figures and Tables

**Table 1:** Subject inclusion/exclusion criteria. Potential subjects, naïve to upper cervical chiropractic care, demonstrated between ten and twenty-six headache days per month self-reported over the previous four months. Requisite was at least eight headache days per month, where intensity reached at least four, on a zero to ten Visual Analog Scale (VAS) pain scale.

Exclusion criteria	Inclusion criteria
Presence of (1) Any medical or psychiatric condition which in the opinion of the screening investigator would make the subject unsuitable for enrolment, because of inability to comply with study requirements or possible confounding of the results (2) More than twenty-six headache days a month (3) Acute medication overuse as defined by the International Classification of Headache disorders (4) Pregnancy or lactation (5) Severe cervical spine degeneration as assessed by cervical spine X-ray (6) Claustrophobia (7) A history of cardiovascular disease, cerebrovascular disease, brain surgery, or other central nervous system disorders (8) Other chronic pain disorders which might interfere with headache assessment or study procedures (9) A history of significant hypo- or hypertension as determined by the investigator (10) Subjects on a beta-blocker, calcium channel blocker, or another medication which the investigator considers might alter cerebral vascular regulation. Triptans are allowed but must not be taken within 24 hours (frovatriptan 48 hours) before a PCMRI study (11) A history of substance abuse or dependence within one year (12) Current participation in a research study or within the last thirty days (13) Any spinal chiropractic care outside of the study protocol is prohibited during the baseline and treatment period	Subject must be or have (1) Male or female, 21 to 65 years of age (2) Signed written informed consent (3) Naïve to upper cervical chiropractic care (4) Migraine with or without aura according to the International Classification of Headache Disorders (5) Had ten to twenty-six headache days per month over the last 4 months (self-reported) (6) At least 4 separate headache episodes per month, with episodes separated by at least 4 hours of pain-free time (7) At least 8 days per month with pain of levels of ≥4/10 for part of the day (8) At least eight headache days per month meeting migraine diagnostic criteria, or where headache is successfully treated with a migraine specific medication (9) Suitable candidates for therapeutic intervention as assessed by NUCCA investigator (10) Subjects on acceptable pharmacological prophylaxis must either remain on a stable dose throughout the study, or stop the prophylactic medication one month before entering the baseline period

**Table 2:** Subject intracranial compliance index (ICCI) data (*n* = 11). PC-MRI^6^ acquired ICCI^1^ data reported at baseline, week four, and week eight following NUCCA^5^ intervention. Bolded rows signify subject with secondary venous drainage route. MVA or mTBI occurred at least 5 years prior to study inclusion, average 10 years.

ID^2^	Age	Sex	Number of NUCCA^5^ corrections	Venous drainage route	ICCI^1^			History of a	
					Baseline	Week four	Week eight	MVA^4^	mTBI^3^
1	34	F	1	Jugular	5.02^*∗*^	5.49	4.79	Yes	Yes
**2**	**44**	**F**	**2**	**Secondary**	**15.2**	**9.29**	**12.98**		**Yes**
**3**	**35**	**M**	**2**	**Secondary**	**4.08**	5.64^*∗*^	**4.86**	**Yes**	**Yes**
4	29	F	1	Jugular	8.73	9.13	9.99		
**5**	**28**	**M**	**2**	**Secondary**	**5.91**	**8.18**	**8.46**	**Yes**	**Yes**
6	43	F	1	Jugular	4.66	4.65	4.63	Yes	Yes
7	47	F	1	Jugular	5.58	4.93	5.59		Yes
8	54	F	3	Jugular	5.04	4.46	3.87^*∗*^		Yes
9	61	F	1	Jugular	5.59	5.66	4.19		Yes
**10**	**52**	**M**	**5**	**Secondary**	**5.7**	**5.3**	13.98^*∗*^	**Yes**	**Yes**
11	20	F	2	Jugular	4.81	6.05	7.14^*∗*^		

^*∗*^Mean of two values provided.

^1^ICCI: intracranial compliance index.

^2^ID: subject identification.

^3^mTBI: mild traumatic brain injury.

^4^MVA: motor vehicle accident.

^5^NUCCA: National Upper Cervical Chiropractic Association.

^6^Phase Contrast Magnetic Resonance Imaging.

**Table 3:** Descriptive statistics [mean, standard deviation, median, and interquartile range (IQR^2^)] of NUCCA^1^ assessments before-after initial intervention (*n* = 11).

NUCCA^1^ assessment	Mean	Standard deviation	Median	Q_1_, Q_3_
Before-NUCCA^1^-Supine Leg Check (inches)	0.73	0.18	0.75	0.5, 0.75
After-NUCCA^1^-Supine Leg Check (inches)	**0.00**	**0.00**	**0.00**	**0, 0**

Before-NUCCA^1^-GSA^3^ Posture Score	31.55	3.91	30.00	28, 35
After-NUCCA^1^-GSA^3^ Posture Score	**3.91**	**1.08**	**3.00**	**3, 4**

Before-NUCCA^1^-Atlas Laterality^*∗*^ (degrees)	3.68	1.57	3.25	2.5, 5.5
After-NUCCA^1^-Atlas Laterality^*∗*^ (degrees)	**1.32**	**1.18**	**0.75**	**0.5, 0.75**

Before-NUCCA^1^-Atlas Rotation^*∗*^ (degrees)	2.57	1.12	3.00	1.5, 3.5
After-NUCCA^1^-Atlas Rotation^*∗*^ (degrees)	0.57	0.85	0.00	0. 1.5

^*∗*^As derived from radiograph measurement.

^1^NUCCA: National Upper Cervical Chiropractic Association.

^2^IQR: interquartile range.

^3^GSA: Gravity Stress Analyzer.

**(a):** 

	Baseline mean (SD)	Week-4 mean (SD)	Week-8 mean (SD)	Difference baseline to week-4 mean (95% CI^1^) *p* value	Difference baseline to week-8 mean (95% CI^1^) *p* value
*Headache diary*					
Headache days per month	14.5 (5.7)	11.4 (5.2)	8.7 (4.3)	3.1 (0.19, 6.0) *p* = 0.039	5.7 (2.0, 9.4) *p* = 0.006
Headache intensity	2.8 (0.96)	2.6 (0.89)	2.1 (1.18)	0.17 (−0.53, 0.86) *p* = 0.604	0.69 (−0.32, 1.71) *p* = 0.158

*Health Related Quality of Life (HRQoL)*					
HIT-6^2^	64.2 (3.8)	55.3 (7.7)	53.8 (6.8)	8.9 (4.9, 13.0) *p* < 0.001	10.4 (6.9, 13.8) *p* = 0.001
					
MSQL-R^6^	38.4 (17.4)	69.1 (22.7)	73.5 (28.0)	30.7 (22.4, 38.9) *p* < 0.001	35.1 (23.5, 46.6) *p* < 0.001
MSQL-E^4^	53.3 (23.5)	82.4 (16.9)	81.2 (29.2)	29.1 (15.9, 42.3) *p* < 0.001	27.9(12.9, 43.1) *p* = 0.002
MSQL-P^5^	54.1 (18.1)	83.2 (16.9)	86.8 (16.9)	29.1 (16.8, 41.4) *p* < 0.001	32.7 (21.3, 44.5) *p* < 0.001

**(b):** 

	Baseline mean (SD)	Week-12 mean (SD)	Difference mean (95% CI) *p* value
MIDAS^7^	46.7 (27.7)	14.6 (23.8)	32.1 (13.2, 51.0) *p* = 0.004

^1^CI: confidence interval.

^2^HIT-6: Headache Impact Test-6.

^3^HRQoL: Health Related Quality of Life.

^4^MSQL-E: Migraine-Specific Quality of Life Measure-Emotional.

^5^MSQL-P: Migraine-Specific Quality of Life Measure-Physical.

^6^MSQL-R: Migraine-Specific Quality of Life Measure-Restrictive.

^7^MIDAS: Migraine Disability Assessment Scale.

**Table 5:** Summary comparison of measured outcomes (*n* = 11).

ID^1^	ICCI^3^	MIDAS^8^	HIT-6^2^	MSQL^4^			Headache diary	
				R^7^	E^5^	P^6^	Days/month	Intensity
1	↔						—	—
2	↓	—	—		—		—	—
3	↑	—						—
4	↑							
5	↑							
6	↔				—			—
7	↔	—						
8	↓							
9	↓	—					—	—
10	↑			—				—
11	↑						—	

↔: remained essentially the same; ↓: decreased; ↑: increased; —: no clinically significant change observed. A blank field in the chart indicates a clinically significant change was observed.

Five subjects demonstrated an increase in ICCI similar to case study results.

Three-subject compliance index remained essentially the same while two showed a decrease. Two subjects showed ICCI increase and a positive change in all HRQoL measures.

^1^ID: subject identification.

^2^HIT-6: Headache Impact Test-6.

^3^ICCI: intracranial compliance index.

^4^MSQL: Migraine-Specific Quality of Life Measure.

^5^MSQL-E: Migraine-Specific Quality of Life Measure-Emotional.

^6^MSQL-P: Migraine-Specific Quality of Life Measure-Physical.

^7^MSQL-R: Migraine-Specific Quality of Life Measure-Restrictive.

^8^MIDAS: Migraine Disability Assessment Scale.

**Table 6:** 24-week intracranial compliance index (ICCI) data (*n* = 2).

ID	Age	Sex	Intracranial compliance index			
			Baseline	Week 4	Week 8	Week 24
01	34	F	5.02	5.49	4.79	6.69
02	44	F	15.17	9.29	12.98	9.47

24-week ICCI findings showing an increasing trend in subject 01 whereas at end of study (week 8), results were interpreted as consistent or remaining the same. Subject 02 continued to show a decreasing trend in ICCI.

## Abbreviations

ASC: Atlas subluxation complex
CHAMP: Calgary Headache Assessment and Management Program
CSF: Cerebrospinal Fluid
GSA: Gravity Stress Analyzer
HIT-6: Headache Impact Test-6
HRQoL: Health Related Quality of Life
ICCI: Intracranial compliance index
ICVC: Intracranial volume change
IQR: Interquartile range
MIDAS: Migraine Disability Assessment Scale
MSQL: Migraine-Specific Quality of Life Measure
MSQL-E: Migraine-Specific Quality of Life Measure-Emotional
MSQL-P: Migraine-Specific Quality of Life Measure-Physical
MSQL-R: Migraine-Specific Quality of Life Measure-Restrictive
NUCCA: National Upper Cervical Chiropractic Association
PC-MRI: Phase Contrast Magnetic Resonance Imaging
SLC: Supine Leg Check
VAS: Visual Analog Scale.
